# Feasibility of Repurposing the Polyanionic Microbicide, PPCM, for Prophylaxis against HIV Transmission during ART

**DOI:** 10.5402/2011/524365

**Published:** 2010-11-28

**Authors:** Robert A. Anderson, David Brown, Erin M. Jackson, Kenneth A. Feathergill, James W. Bremer, Ralph Morack, Richard G. Rawlins

**Affiliations:** ^1^Department of Obstetrics and Gynecology, Rush University Medical Center, 1653 W. Congress Parkway, Chicago, IL 60612, USA; ^2^AndroJek, Inc., 2145 W. Davie boulevard, Suite 103, Ft. Lauderdale, FL 33312, USA; ^3^Department of Immunology and Microbiology, Rush University Medical Center, 1653 W. Congress Parkway, Chicago, IL 60612, USA

## Abstract

HIV-serodiscordant couples wishing to conceive often seek assisted reproduction, during which spermatozoa from infected men are washed to minimize the risk of HIV transmission to partner and fetus. We sought to improve this method by adding a microbicide, PPCM, as an HIV prophylactic. HIV-1 (BaL) inhibition by PPCM appears irreversible and independent of added Ca^2+^. Without added Ca^2+^, PPCM (≤10 mg/mL, ≤90 min), a stimulus of Ca^2+^-dependent acrosomal loss, has no effect on sperm motility, forward progression, or acrosomal status. PPCM-treated (10 mg/mL) sperm retain their ability to acrosome react when Ca^2+^ is added. Sperm DNA integrity/function is unaffected by PPCM (≤10 mg/mL). Adding PPCM (5 mg/mL, 30 min) to washing media reduces infectivity (viral antigen p24 and RNA) of *ex-vivo* HIV-infected semen by 3-4 Logs compared with washing alone. Sperm washing with appropriate extracellular Ca^2+^ levels and PPCM is significantly more effective than washing alone at reducing HIV infectivity.

## 1. Introduction

There were approximately 33.4 million people worldwide living with HIV in 2008; of these, 2.7 million were newly infected. There were approximately 2.0 million AIDS-related deaths in 2008 [1]. These statistics are substantially below those seen during the apparent peak in infections in 1996. At least part of the reason for the trend toward stabilization of the AIDS epidemic and increased longevity of HIV-infected individuals can be attributed to the beneficial effect of antiretroviral therapy [1]. 

 Decreased risk of viral transmission and the prospect of increased longevity of the infected parent secondary to the use of highly effective antiretroviral therapy pose fewer challenges for infected individuals of reproductive age to have their own children. However, HIV infection may impair fertility [2–4], prompting HIV-infected men and women to seek medical assistance (i.e., Assisted Reproductive Technologies, or ART). Further, ART is the method of choice for fertile serodiscordant couples trying to conceive; it reduces the risk of HIV transmission associated with unprotected sexual intercourse [5].

 Providing ART to HIV-infected couples, particularly serodiscordant couples where the man is infected, is problematic for health care providers. HIV is sometimes detected in semen from infected men, even when they are receiving antiretroviral therapy [6, 7]. Spermatozoa contaminated with HIV can transmit virus to the uninfected woman and to the child [2, 8]. Several states in the U.S. prohibit use of semen from HIV-infected individuals for insemination [9].

 Sperm washing has been advocated as a method to reduce HIV transmission, in which motile spermatozoa are separated from seminal plasma, nonsperm cells, and HIV [9, 10]. This method appears to be successful, with no reported infections in multiple procedures carried out in somewhat more than 2000 couples [11, 12]. This was, in large part, the basis for California legislation that permits a sperm wash to minimize HIV transmission during ART procedures for serodiscordant couples [13, 14].

 However, not all infected semen processed by sperm washing can be used. Due to the nature of the sperm washing method, detectable HIV titers remain in 4–6% of washed sperm samples [4, 12, 15, 16]. Reasons for this are not entirely clear, but may be due, at least in part, to viral binding to the sperm fraction.

For the most part, neither conventional HIV-1 CD4 receptors nor coreceptors (CXCR4 and CCR5) have been found on human spermatozoa [10, 17, 18]. However, several studies [17, 19, 20] have shown that HIV binds to spermatozoa. Binding appears avid, since it survives repeated washes by gradient centrifugation. Sperm-bound HIV is infectious [17, 20]. 

 Alternative receptors had been proposed to account for HIV binding [19, 21]. Ceballos and coworkers found that HIV-1binds to human spermatozoa through sperm surface heparan sulfate receptors and remains infectious [20].

 HIV binding, through alternative receptors or nonspecific mechanisms, may explain the failure of a sperm wash to completely remove HIV from infected semen in all instances. Further, at least some proponents of sperm washing to reduce risk of HIV transmission during assisted reproduction advise against its use with intracytoplasmic sperm injection (ICSI). This recommendation is based on the possibility that ICSI may introduce HIV into the oocyte as a result of HIV attached to the sperm membrane [4, 22].

Addition of a safe antiviral compound may offer some advantages over sperm washing alone in minimizing the risk of HIV transmission during ART. Ideally, an effective antiviral agent added at or around the time of sperm washing should result in the following: (1) processed sperm samples would contain no residual infectious HIV; (2) ICSI could be used as an assisted reproductive technique in HIV-serodiscordant couples with fewer concerns; (3) treatment could be offered to a larger number of serodiscordant couples than possible with the conventional sperm wash protocol, which is biased in favor of very low or undetectable initial viral load [8, 12]; (4) the risk of viral infection of health care providers who are in direct contact with infected samples would be greatly reduced.

 Non-antiretroviral compounds have been developed, intended for vaginal use, to prevent HIV transmission during sexual intercourse. These compounds, known generically as microbicides, have advanced up to and including clinical trials [23–26]. Many belong to a class of compounds known as polyanions, one of which is a poly-acidic oligomer, code-named PPCM (formerly known as Sulfuric Acid-Modified Mandelic Acid, or SAMMA), developed [27–32] by the TOPCAD Program at Rush University Medical Center (Chicago, IL) and licensed by Yaso Biotechnologies (Phoenix, AZ).

 Polyanions tested to date, including PPCM, are noncytotoxic, broad-spectrum antimicrobial agents, with activities against sexually transmitted pathogens, including HIV-1, HSV-1, HSV-2, papillomavirus, *N. gonorrhoeae,* and *C. trachomatis* [27–29, 31, 33]. They act in part by interfering with microbial binding to target cells via the heparan sulfate (or similar) receptor [34]. PPCM may be, therefore, an attractive candidate as a safe antiviral agent that could be used as an HIV-1 preventive agent during ART.

 However, many polyanions, including PPCM, are contraceptive [34–37]. These compounds would be appropriate for ART only if their antimicrobial activities can be separated from their contraceptive activities, and they have no adverse effects on sperm function and integrity. 

 Contraceptive activities of polyanions may be due, at least in part, to their abilities to induce premature acrosomal loss. Mechanistic studies have identified this as a Ca^2+^-dependent process [30]. We thought that it may be possible to separate contraceptive and antimicrobial activities of PPCM through manipulation of extracellular Ca^2+^ levels.

 The purpose of the present study was to examine the feasibility of the use of PPCM to reduce infectivity of HIV-1 in infected semen used for ART. Results favor the utility of PPCM for this purpose. Inhibition of HIV-1 (BaL) by PPCM is at least partially irreversible and is independent of added extracellular Ca^2+^. In the absence of added Ca^2+^, sperm washing in the presence of PPCM substantially reduces (3-4 Logs) viral titer of *ex-vivo* HIV-1 (BaL)-treated whole human semen from healthy donors, as compared with washing alone. This occurs without negatively affecting measures of sperm DNA integrity or sperm function, including motility, forward progression, acrosomal integrity, and DNA decondensation.

## 2. Materials and Methods

### 2.1. Materials

ISolate, a colloidal density gradient medium for separation of spermatozoa from semen for use in ART, was purchased from Irvine Scientific (Santa Ana, CA). Quinn's sperm wash medium was purchased from SAGE In-Vitro Fertilization, Inc. (Trumbull, CT). RPMI-1640 medium (RPMI) was from Irvine Scientific; a custom preparation of RPMI to which Ca^2+^ had not been added was also provided by Irvine Scientific. HIV-1 (BaL; lot # 07283002), an expanded laboratory strain, was from the Virology Quality Assessment Program (VQA), Rush University Medical Center and was provided by the Clinical Retrovirology Research Laboratory, Department of Immunology and Microbiology, Rush University Medical Center, under the direction of Dr. J. Bremer. Reagents for immune complex disruption and ELISA determination of HIV-1 antigen p24 (hereafter referred to as p24) levels, and real-time reverse transcription-PCR determination of HIV-1 RNA were obtained in kit forms from PerkinElmer Life Sciences, Inc. (HIV-1 p24 ELISA Kit; Boston, MA) and Abbott Molecular, Inc.(RealTime HIV-1 RNA assay; Des Plaines, IL; m2000 RealTime-PCR-System), respectively. Calcium ionophore A23187 was from EMD Biosciences, Inc. (Calbiochem; La Jolla, CA). All other reagents were of the highest quality commercially available.

PPCM is licensed under U.S. Patent 5,932,619 by Yaso Biotechnologies (Phoenix, AZ). It is a poly-acidic oligomer with molecular weight of approximately 1500 Da and was synthesized by Dr. S. Jain, University of Illinois Health Sciences Center (Chicago, IL) under the direction of the TOPCAD Program by reacting D,L-mandelic acid with sulfuric acid under proprietary conditions. The sodium salt was prepared by reacting the free acid with alcoholic NaOH, yielding an off-white powder.

### 2.2. Human Subjects

Semen samples used for HIV infectivity, sperm motility, and acrosomal status assessment were collected from pools of eight healthy donors (Rush University Medical Center) and five fertile donors (Andromedix Laboratory) from whom informed consent was given, according to the protocols approved by their respective Institutional Review Boards. For semen used in all experiments except DNA testing the mean donor age was 35 (range = 26–44) years. Semen was of generally high quality; mean semen volume was 3.0 ± 0.24 (standard error of the mean; SEM, *N* = 13) mL. Initial mean (with 90% confidence limits) values for sperm count, % motility, % forward progression, and % viable spermatozoa were 83 (58.9–117.8; *N* = 11) × 10^6^/mL, 69 (64.3–73.6; *N* = 12)%, 78 (72.0–82.9; *N* = 12)% and 90 (85.7–93.0; *N* = 12)%, respectively.

Semen used for SDFA and SDD testing was derived from five donors of proven fertility, selected for the ability of their spermatozoa to undergo DNA decondensation (96% ± 2% SD) within 15 minutes of adding stimulus.

### 2.3. Semen Collection and Processing

Semen was collected into sterile containers by self-masturbation. Samples were allowed to stand at ambient temperature for one hour to allow for liquefaction. Spermatozoa were separated from seminal plasma by centrifugation through a discontinuous gradient consisting of equal volumes of 45% and 90% ISolate (Irvine Scientific) at 200× g for 20 min. Spermatozoa at the bottom of the gradient were resuspended in Quinn's Sperm Wash and washed by centrifugation at 200× g for 10 min. Unless otherwise noted, the sperm pellet was suspended in medium (either BWW or RPMI), to which Ca^2+^ had not been added, prior to further use.

### 2.4. Sperm Viability/Function Variables

#### 2.4.1. Motility, Forward Progression, and Viability

Motility measurements of 10 *μ*L samples were carried out under light microscopy (200x) with a Neubauer hemacytometer by standard operating procedures used by the Andrology Laboratory of the Rush Centers for Advanced Reproductive Care, Rush University Medical Center. Motile spermatozoa were defined as cells displaying any type (progressive, in-place) of movement; spermatozoa passing through the field of vision were considered forwardly progressing. From 200–400 spermatozoa were examined in at least 10 nonadjacent fields. Motility was recorded as percentage of total spermatozoa that were moving; forward progression was recorded as the percentage of motile spermatozoa that was forwardly progressive.

 Sperm viability was measured with Eosin Y. Staining was visualized by bright field light microscopy (400x). Spermatozoa that stained green were considered viable; spermatozoa that stained red were regarded as nonviable. Results were recorded as the percentage of total spermatozoa that were viable. Results for sperm motility, forward progression and viability are presented as mean percentage of postwash control values, together with SEM.

#### 2.4.2. Acrosomal Status and Acrosomal Loss

Within the context of this study, acrosomal loss refers to the disruption of the sperm acrosome in response to a treatment or chemical entity. No inference is made as to whether this response is identical to a physiological acrosome reaction, during which the acrosome is also lost.

 Spermatozoa recovered from ISolate gradient centrifugation were resuspended in modified Biggers, Whitten and Whittingham (BWW; [38]) medium (less albumin and Ca^2+^). After incubations of washed spermatozoa with 10 mg/mL PPCM or with medium alone for 15 min at ambient temperature, samples were placed onto 1 mL of 11% (wt/vol) buffered Ficoll (containing 120 mM NaCl and 25 mM HEPES, pH 7.4), and centrifuged at 15,000 g-min (22°C to 24°C). Supernatants were aspirated from the sperm pellets, and the pellets were resuspended into modified BWW medium. The suspensions were recentrifuged at 1,000 g-min. The supernatants were aspirated, and the pellets were resuspended in 1 mL BWW medium. The latter washing step was repeated, at which point, the PPCM concentration in the supernatant, as measured by the absorbance at 266 nm, was undetectable. Details of sperm preparation have been described [39, 40].

 Sperm pellets obtained from the above procedure were resuspended in BWW medium that contained 2.3 mM CaCl_2_. After equilibration for 10 min at 37°C, acrosomal loss was induced by addition of stimulus (PPCM or calcium ionophore A23187). Fifteen min after acrosomal loss induction, spermatozoa were fixed with buffered glutaraldehyde, smeared onto slides and stained with Rose Bengal and Bismarck brown for acrosome visualization [30, 39]. Approximately 450 cells were scored per slide. Data are expressed as means, with 90% confidence limits, of percentage of acrosomal loss induced.

#### 2.4.3. Sperm DNA Fragmentation Assay (SDFA) and Sperm DNA Decondensation (SDD) Test

The SDFA and SDD Test were carried out at the Andromedix Laboratory, Woburn, MA. The SDD Test was formerly known as the Human Sperm Activation Assay (HSAA, [41]).

 Experiments were conducted at ambient temperature. Fresh semen samples were divided into aliquots as follows.

**Controls**

Semen was mixed with 2 mL “nuclear isolation medium” (NIM), consisting of 200 mM sucrose, 2.4 mM MgCl2, 10 mM Tris-HCl, 5 mM maleic acid, pH 7.4, and centrifuged at 1200× g for 10 min to remove seminal plasma. Sperm pellets were resuspended in 1.0 mL of NIM (solvent for PPCM), prior to the SDFA, or analysis in the SDD Test as described by Brown et al. [41].

**Treated Samples**

Sperm pellets were resuspended in 1.0 mL of NIM containing PPCM (2 mg/mL or 10 mg/mL, final concentrations). After a 10 min incubation, samples were centrifuged at 1200× g for 5 min; sperm pellets were resuspended in 1.0 mL NIM and the suspension was recentrifuged. The final pellets were either resuspended in 0.5 mL NIM and snap-frozen in liquid nitrogen prior to the SDFA, or resuspended in 1.0 mL NIM and kept overnight at 4°C prior to analysis in the SDD Test.

#### 2.4.4. SDFA

The SDFA uses the same methodology as the Sperm Chromatin Structure Assay [42]. The SDFA utilizes Acridine Orange (AO) as a fluorescent DNA probe and employs flow cytometry to measure the intensity of fluorescence. AO is a cell-permeant nucleic acid binding dye that emits green fluorescence when bound to double-stranded (normal) DNA and red fluorescence when bound to single-stranded (damaged) DNA. For each sample, 5000 cells were measured and the ratio of red to total fluorescence (red + green) was calculated. This value, expressed as a percentage, is the DNA Fragmentation Index (DFI). Each assay was carried out in duplicate, which was averaged. All averaged data for each semen donor were subjected to arcsine transformation prior to further analysis. Values are given as means, with 90% confidence limits. A DFI score of ≥30 indicates significantly compromised DNA integrity. For this study, DFI scores ≤15 were considered normal. A mean score where 15 < DFI < 30, suggests a marginal loss of DNA integrity due to treatment.

#### 2.4.5. SDD Test

Samples (see above) were centrifuged at 1200× g for 10 min and resuspended in permeabilization medium for 5 min. They were sequentially washed with NIM containing bovine serum albumin, and treated for one hour with Xenopus egg extract isolation medium (XEIM; 10 mM Tris-HCl, 1.5 mM MgCl_2_, 100 mM KCl, 50 mM dithiothreitol, pH 7.5). Sperm suspensions were centrifuged at 1200× g for 10 min, and the pellets were suspended in XEIM to a final cell concentration of 25 × 10^6^ cells/mL. For each sample, 50,000 permeabilized sperm were incubated in 25 *μ*L of *Xenopus laevis* egg extract, to simulate the *in vivo* environment of the sperm nucleus postfertilization. After 15 min, an aliquot of the sperm-extract mixture was placed on a slide, and 50–100 sperm were scored with phase-contract microscopy in real time during a 5 min window. The percentage of sperm undergoing full decondensation was determined. The measurement of full decondensation has been fully described by Brown et al. [41]. Data were processed as described for the SDFA. Values are presented as mean % fully decondensed spermatozoa, with 90% confidence limits.

### 2.5. HIV-1 Infectivity

#### 2.5.1. Inhibition of HIV-1 (BaL) by PPCM: Effect of Added Extracellular Ca^2+^

PPCM at several concentrations ranging from 0.1 to 20 mg/mL was added to HIV-1 (BaL) suspensions (5.9 × 10^3^  TCID_50_s/mL). Medium was RPMI-1640, supplemented with glutamine, gentamycin, human growth factor and interleukin-2, with or without added Ca^2+^ (0.46 mM Ca(NO_3_)_2_–4(H_2_O)). After 1 h at ambient temperature, suspensions were diluted 1 : 100 with RPMI (with or without added Ca^2+^), prior to inoculation into peripheral blood mononuclear cell (PBMC) suspensions (2.0 × 10^6^ cells in 0.1 mL). After 1 h, PBMCs were washed with fresh medium (containing added Ca^2+^) to remove unbound virus and PPCM, and the PBMC suspensions were incubated for 72 hours at 37°C to allow for viral replication. 

Viral p24 levels were measured by ELISA with reagents provided in kit form by PerkinElmer Life Sciences, Inc., according to instructions provided by the vendor. Values were quantified with a standard curve of HIV-1 p24 concentration as a function of absorbance at 490 nm. Intra- and interassay coefficients of variance are approximately 2.0% and 10.0%, respectively. Data are expressed as means ± SEM. The concentration of PPCM required for 50% inhibition of p24 production (IC_50_ value, obtained from curves fit to the data with TableCurve 2D; see Statistical Analysis) when PPCM was added to complete RPMI was compared with that obtained when PPCM was added to medium without added Ca^2+^.

#### 2.5.2. Reversibility of HIV-1 Inhibition by PPCM

Inhibition of viral replication by PPCM was compared under two conditions, as follows: (1) PPCM at concentrations up to 13 mg/mL was added to only HIV-1 (BaL), and incubated for 1 h, followed by a 1 : 100 dilution in RPMI, prior to the suspension being inoculated onto PBMCs. Subsequent washing medium changes and incubation of PBMCs to allow for viral replication were carried out in the absence of PPCM. (2) PPCM at concentrations up to 1 mg/mL was added to viral suspensions, incubated for 1 h, and inoculated onto PBMCs that contained the same concentrations of PPCM. Washing and medium changes also contained PPCM (i.e., PPCM was present throughout the entire procedure). The incubation medium (RPMI) contained added Ca(NO_3_)_2_–4(H_2_O). Other experimental conditions and measurement of p24 levels were the same as described above for effect of added extracellular Ca^2+^. The IC_50_ value of PPCM when PPCM was added to only virus was compared with that obtained when PPCM was present throughout the viral infection and replication.

#### 2.5.3. Inhibition by PPCM of HIV-1 Infectivity When HIV Is Added to Whole Semen

Neat fresh semen pooled from five donors (11.3 mL) was added to an equal volume of HIV-1 (BaL) inoculum (5 × 10^5^  TCID_50_s/mL). Total inoculum and total number of spermatozoa were 5.65 × 10^6^   TCID_50_s and 1.07 × 10^9^ cells, respectively. After 30 min at ambient temperature, small samples were taken, diluted and added directly to PBMC suspensions (unprocessed control). One volume of either 0.9% saline (processed control) or PPCM stock solution (20 mg/mL in 0.9% saline, sterile-filtered) was added to three volumes of the semen/viral suspension. These mixtures were incubated for 45 min at ambient temperature. Spermatozoa were isolated by centrifuging the mixture through sperm ISolate preparation gradients (200× g, 25 min). Gradients were aspirated. The sperm pellets and residual supernatants (total volume approx. 0.2 mL) were resuspended in 1 mL RPMI without added Ca^2+^ and centrifuged at 200× g for 10 min. Sperm pellets (approximately 0.2 mL) were used as the viral inoculum, and added to PBMC suspensions. After 1 h, PBMCs were washed to remove unbound virus, spermatozoa and PPCM, and incubated for 72 h in Ca^2+^-replete RPMI to permit viral replication. At this time, a portion of each sample was taken for p24 measurement (see above).

 The remainder of each sample was stored at −80°C until being used for viral RNA measurements by real-time reverse transcription PCR (m2000 Real Time HIV assay), with automated RNA preparation (m2000sp) and measurement (m2000rt) carried out as instructed by the manufacturer. The assay was standardized against a viral standard from the VQA Program (see Materials). The assay is designed to achieve an interassay standard deviation of ≤0.25 log copies/mL at concentrations ranging from 500 to 5 × 10^6^ copies/mL. Data (copies/mL viral RNA) are expressed as means ± SEM.

### 2.6. Statistical Analysis

For data requiring either logarithmic (sperm count) or arcsine transformation (frequencies; initial sperm motility, forward progression, viability; acrosomal loss), results are expressed as means, with 90% confidence limits [43]. Other data are expressed as means, ± SEM.

 Effects of PPCM dose and duration of exposure were evaluated with two-way analysis of variance. Differences among individual treatment groups were assessed with the Newman-Keuls multiple range test [44]. Data from dose response experiments were best fit to curves by regression analysis with TableCurve 2D curve-fitting software (version 5; SPSS Statistical Software, Chicago, IL), from which appropriate constants (e.g., IC_50_; that concentration of agent required for 50% inhibition of HIV infectivity) were calculated. All values are considered significantly different at the 0.05 level of confidence; *P* > .10 is considered not different.

## 3. Results

### 3.1. Inhibition of HIV Infectivity by PPCM Is Independent of Added Ca^2^

**Figure 1:**
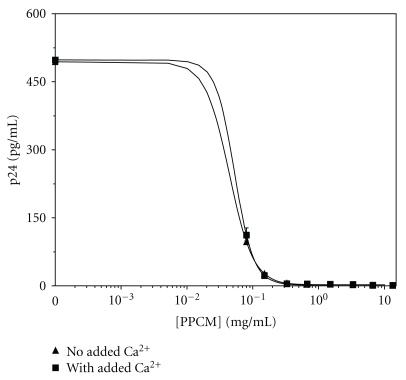
Inhibition of HIV-1 BaL by PPCM is independent of added Ca^2+^. Values are levels of p24 (pg/mL); Errors are SEM (*N* = 3). Media contained 100 mg/mL Ca(NO_3_)_2_–4(H_2_O), either throughout the procedure (added Ca^2+^) or only after washing PBMCs to remove unbound virus and PPCM (i.e., during PBMC incubation to allow for viral replication). Data were fit to transitional curves described by the general equation for a log-normal cumulative distribution, *Y* = *a* + 0.5*b*(*erf*)*c*(−ln (*x*/*c*)/(2^0.5*d*^)). In the absence of added Ca^2+^, *a* = 2.38408, *b* = 491.6185, *c* = 0.03921, and *d* = −0.82000 (*r* ^2^ = 0.9999); in the presence of added Ca^2+^, *a* = 0.00853, *b* = 497.99248, *c* = 0.02378, and *d* = −0.76421 (*r* ^2^ = 0.9995). The IC_50_ values of PPCM with and without added Ca^2+^ are 24 (90% confidence limits = 33.7–66.3% inhibition) and 39 (46.8–53.2% inhibition) *μ*g/mL, respectively.

Dose responses of HIV infectivity as a function of PPCM concentration (from 1 × 10^−5^ to 6.7 mg/mL) were measured in the presence and absence of added Ca^2+^. In these experiments, all samples contained Ca^2+^ during the 72 h incubation of PBMCs to allow for viral replication of infected cells. Ca^2+^ is essentially without effect on the ability of PPCM to reduce HIV infectivity. The IC_50_ (90% confidence limits = 33.7–66.3% inhibition) for samples incubated in Ca^2+^-replete RPMI is 24 *μ*g/mL. In Ca^2+^-deficient medium, the IC_50_ (46.8–53.2% inhibition) is 39 *μ*g/mL (Figure 1).

### 3.2. HIV Inhibition by PPCM Is Not Reversible by Dilution

Dose response of HIV infectivity as a function of initial PPCM concentration (from 1 × 10^−5^ to 13.3 mg/mL) was measured in samples in which PPCM concentrations were maintained throughout the experimental procedure (viral incubation, inoculation onto PBMCs, wash to remove free virus and incubation of infected PBMCs to permit viral replication). This was compared with the dose-response measured in samples in which PPCM-treated virus was first diluted 1 : 100 prior to inoculation onto PBMCs. In the latter samples, medium used for procedures subsequent to initial viral exposure to PPCM was PPCM-free.

**Figure 2:**
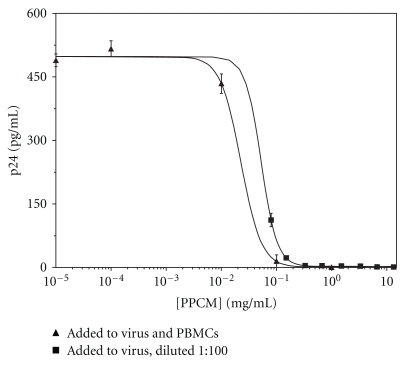
Evidence for irreversibility of HIV-1 BaL by PPCM. PPCM was added to either virus and PBMCs (present during viral infection and replication) at the indicated concentrations, or only to virus, followed by a 1 : 100 dilution prior to inoculation into PBMC suspensions. Other conditions are presented in Section 2. Values are levels of viral p24 (pg/mL). Errors are SEM. For incubations in which PPCM was present throughout the experiment at the indicated concentrations, data were fit to transitional curves described by the general equation for a log-normal cumulative distribution, *Y* = *a* + 0.5*b*(*erf*)*c*(−ln (*x*/*c*)/(2^0.5*d*^)); *a* = 0.00853042, *b* = 497.99247, *c* = 0.023779, and *d* = −0.764206 (*r* ^2^ = 0.9995). For incubations in which PPCM was added only to virus, followed 1 h later by a 1 : 100 dilution before inoculation into PBMC suspensions, data were fit to a curve described by the equation *Y* = *a*exp (−*x*/*b*), where *a* = 498.29049 and *b* = 0.0526604 (*r* ^2^ = 0.9995). IC_50_ values of PPCM when added only to virus and when present throughout the procedure were 36 (48.6–51.4% inhibition) and 23 (33.7–66.3% inhibition) *μ*g/mL, respectively.

Dilution and subsequent removal of PPCM by washing has essentially no effect on the ability of PPCM to inhibit HIV infectivity (Figure 2). When the initial concentration of PPCM is maintained throughout all procedures (no dilution), the IC_50_ (33.7–66.3% inhibition) for PPCM is 23 *μ*g/mL. The IC_50_ (90% confidence limits = 48.6–51.4% inhibition) for PPCM when added only to viral suspensions, followed by dilution, is 36 *μ*g/mL.

### 3.3. PPCM Has Minimal Effect on Sperm Motility and Viability

**Figure 3:**
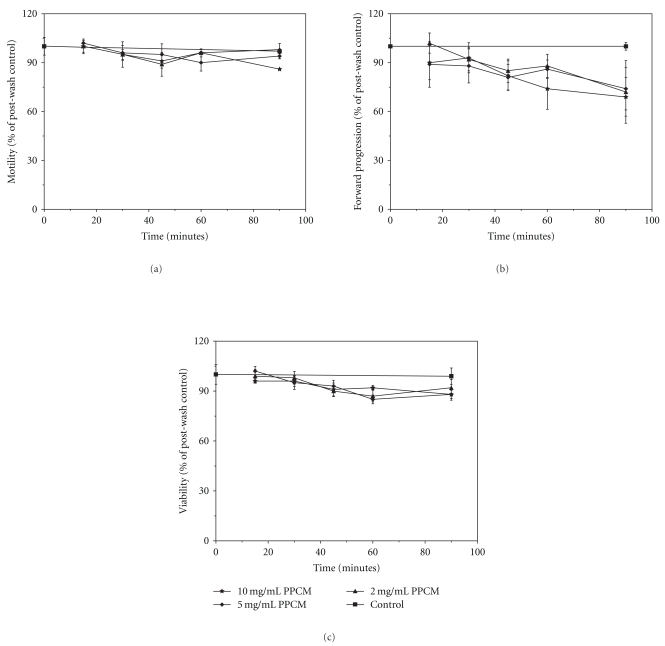
Lack of effect of PPCM on sperm motility, forward progression and viability. Washed spermatozoa were exposed to PPCM at different concentrations and durations of exposure, as indicated. The experimental protocol for these experiments is presented in Section 2. (a): Neither concentration nor duration of treatment with PPCM affects sperm motility. Values are the means ± SEM of duplicate determinations from each of 3 donors (number of donors represents sample size). Two-way ANOVA shows no effect of either duration of treatment (*F*(4,30) = 1.43; *P* > .1) or dose of PPCM (*F*(2,30) = 1.36; *P* > .1) after exposure to 10 mg/mL PPCM for up to 90 min. Similarly, one-way ANOVA shows no overall treatment effect of PPCM (*F*(15,32) = 0.99; *P* > .1). (b): Neither concentration nor duration of exposure affects sperm forward progression. Values are the means ± SEM of duplicate determinations from each of 3 donors (number of donors represents sample size). Two-way ANOVA shows no effect of either duration of treatment (*F*(4,30) = 1.63; *P* > .1), or concentration of PPCM (*F*(2,30) = 1.48; *P* > .10). Similarly, one-way ANOVA shows no effect of treatment (*F*(15,32) = 0.85; *P* > .1). (c): Duration of exposure to, but not dose of PPCM has small effect on sperm viability. Data are expressed as percentage of postwash control viability, as described in Section 2. Values are the means ± SEM of duplicate determinations from each of 3 donors (number of donors represents sample size). Two-way ANOVA shows an effect of duration of treatment (*F*(4,30) = 5.03; *P* < .005), but not concentration of PPCM (*F*(2,30) = 1.67; *P* > .1); no interactive effect of dose and exposure time is seen. The Newman-Keuls multiple range test indicates essentially no effect among treatment groups (*P* > .05). One-way ANOVA shows an overall effect of treatment (*F*(15,32) = 2.054; *P* < .05). Viability at 90 min for samples exposed to either 5 mg/mL or 10 mg/mL PPCM is reduced by 11% as compared with the untreated sample at 90 min (*P* < .05, Newman-Keuls multiple range test).

Sperm exposure to PPCM at concentrations up to 10 mg/mL for periods up to 90 min has little effect on sperm motility, forward progression or viability (Figures 3(a)–3(c)). Two-way ANOVA showed no effect of either PPCM concentration or duration of exposure to PPCM on motility (*F*(2,30) = 1.36 and *F*(4,30) = 1.43, resp.; *P* > .10) or forward progression (*F*(2,30) = 1.48 and *F*(4,30) = 1.63, resp.; *P* > .10). Similarly, there is no effect of PPCM concentration on sperm viability (*F*(2,30) = 1.67, *P* > .10), although a small effect (11% reduction at 90 min exposure to 10 mg/mL PPCM) of duration may exist (*F*(4,30) = 5.03, *P* < .01).

### 3.4. Acrosomal Status Maintained after Sperm Treatment with PPCM in Absence of Added Ca^2+^

**Figure 4:**
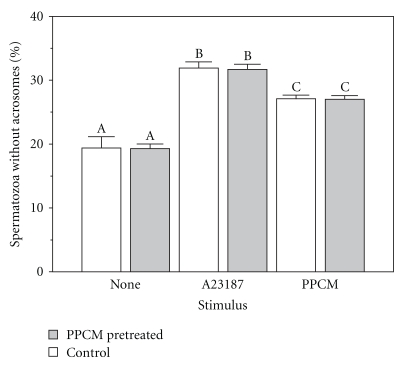
Acrosomal status and ability to respond to acrosomal loss stimuli are maintained after PPCM pretreatment. Motile spermatozoa harvested from pooled semen were resuspended in Ca^2+^-deficient medium and treated with 10 mg/mL PPCM for 15 min, followed by gradient and differential centrifugation to reduce PPCM concentration to undetectable levels. Spermatozoa were resuspended in Ca^2+^-replete medium and acrosomal status was evaluated before and after adding stimuli (PPCM or A23187) of acrosomal loss. Experimental details are provided in Section 2. Values are given as mean percentage of spermatozoa lacking acrosomes; error bars are 90% confidence limits. ^A-C^Values with different letter designations are different (*P* < .001, Newman-Keuls multiple range test).

Acrosomal status of sperm suspensions treated with 10 mg/mL PPCM for 15 min in BWW medium without added Ca^2+^ is similar to control spermatozoa (no PPCM treatment) similarly processed, when spermatozoa from both groups are subsequently washed and added to Ca^2+^-replete medium (detailed protocol presented in Section 2). Percentage of PPCM-treated spermatozoa without acrosomes is 19 (90% confidence limits = 18.6–20.0)%. This is essentially the same as the percentage (19 (17.7–21.2)%) of control spermatozoa without acrosomes (*P* > .10, Newman-Keuls multiple range test). Acrosomal loss increases to a similar extent in both PPCM-treated and control samples in response to stimuli (*P* > .10, Newman-Keuls multiple range test). Percentage of spermatozoa without acrosomes in response to a maximally stimulating concentration of calcium ionophore A23187 is 32 (31.0–32.5)% and 32 (30.9–32.9)% for PPCM-pretreated and control spermatozoa, respectively. Percentage of spermatozoa without acrosomes in response to 0.25 *μ*g/mL PPCM (concentration required to induce 50% maximal acrosomal loss [30]) is 27 (26.3–27.6)% and 27 (26.6–27.7)% for PPCM-pretreated and control spermatozoa, respectively (Figure 4).

### 3.5. PPCM without Effect on Sperm DNA Fragmentation or Stimulus-Induced DNA Decondensation

**Figure 5:**
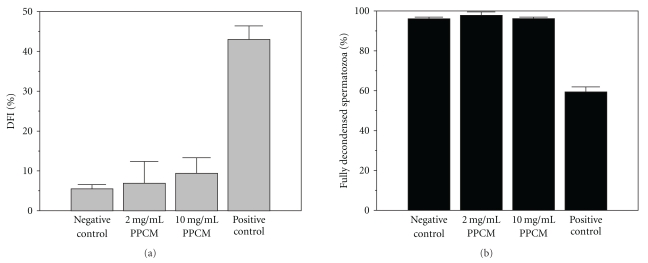
PPCM is without effect on sperm DNA fragmentation and decondensation of sperm DNA in response to stimulus. The sperm DNA fragmentation assay (SDFA) and sperm DNA decondensation (SDD) test were carried out as described in Section 2. (a)**:** Data for the SDFA were expressed as percentage Defragmentation Index (DFI), defined as the ratio of red fluorescence to total fluorescence (red plus green) after staining with acridine orange. Processed samples to which PPCM were not added are designated as Negative Controls. Data for a semen sample with a known high level of sperm DNA fragmentation are included as a reference, designated as Positive Control. Values are given as means; error bars represent 90% confidence limits. One-way ANOVA (excluding the Positive Control) showed no effect of treatment (*F*(2,9) = 2.80; *P* > .10). (b): For the SDD test, fully decondensed spermatozoa are presented as a percentage of total spermatozoa. Error bars are 90% confidence limits (*N* = 3–5). Samples to which PPCM were not added are designated as Negative Controls. Data for a semen sample with a known compromised DNA decondensation are included as a reference; designated as Positive Control One-way ANOVA (excluding the Positive Control) shows no effect of PPCM treatment on sperm DNA decondensation (*F*  (2,8) = 0.59; *P* > .1).

Pretreatment of spermatozoa with either 2 mg/mL or 10 mg/mL PPCM had no effect on sperm DNA fragmentation (Figure 5(a)). Fragmentation in spermatozoa pretreated with PPCM is essentially the same as that in spermatozoa pretreated with only medium (*F*(2,9) = 2.80; *P* > .10). Mean DFI for negative control spermatozoa, spermatozoa treated with 2 mg/mL PPCM and spermatozoa treated with 10 mg/mL PPCM are 6 (90% confidence limits = 4.5–6.6), 7 (2.9–12.4) and 9 (6.1–13.3), respectively.

 A similar lack of effect of PPCM was noted on the ability of DNA from permeabilized spermatozoa to decondense in response to *Xenopus laevis* egg extract (*F*(2,8) = 0.59; *P* > .10). Mean percentage of control spermatozoa, spermatozoa pretreated with 2 mg/mL PPCM, and spermatozoa pretreated with 10 mg/mL PPCM that showed full decondensation in response to egg extract are 96 (95.0–97.0)%, 98 (87.2–99.5)%, and 96 (95.5–96.9)%, respectively (Figure 5(b)).

### 3.6. Infectious Viral Titer of Whole Semen with Simulated HIV Infection Is Reduced by PPCM

Following incubation with HIV-1 (BaL) for 30 min, whole semen was treated with either 5 mg/mL PPCM or with an equal volume of medium (without added Ca^2+^), and incubated at ambient temperature for 45 min (see Section 2 for complete description of experimental protocol). In samples without PPCM, gradient centrifugation, followed by two washes by resuspension and recentrifugation, reduced the initial viral titer by approximately 92%, as measured by viral p24 levels. A much greater reduction in viral titer is effected by pretreatment with PPCM. PPCM further reduces viral p24 levels by approximately 3 Logs, (99.94% reduction, to 0.04 ± 0.03 (SEM) ng/mL) as compared with samples subjected to only centrifugation.

**Figure 6:**
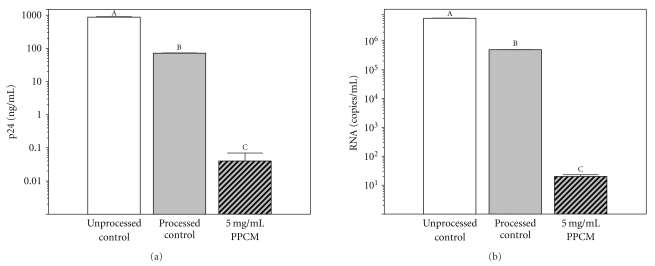
PPCM decreases infectivity of semen infected *in vitro* with HIV. Details of treating whole semen with HIV-1 (Bal) followed by PPCM exposure and measurement of viral titer after culturing in PBMCs are presented in Section 2. Data are reported as mean viral titer (ng/mL of p24, (a): or copies/mL viral RNA, (b)) for each condition (note Log scale). Error bars are SEM (*N* = 3). ^A-C^Values with different letter designations are different (*P* < .01, Newman-Keuls multiple range test).

 Greater reduction of viral titer by PPCM is obtained when titer is expressed as viral RNA (Figure 6(b)). Similar to p24 concentrations, gradient centrifugation followed by washing (without PPCM) reduces viral RNA concentration by 92% (to 4.91 ± 0.055 × 10^5^ copies/mL). Viral RNA is further reduced by approximately 4.4 Logs (to 20 ± 3 copies/mL) by pretreatment with PPCM.

 Reductions in viral titer from that observed in the unprocessed, HIV-incubated semen produced by either sperm washing or PPCM pretreatment followed by sperm washing are significant (*P* < .01, Newman-Keuls multiple range test). Differences are significant whether viral p24 or viral RNA concentration is the dependent variable.

## 4. Discussion

The present data support the utility of PPCM as an effective compound for use in ART to further reduce the risk of HIV transmission. Data collected in other investigations for this and similar compounds suggest that PPCM should be relatively safe for use in ART, and should be efficacious in reducing infectivity of HIV (laboratory strains, clinical isolates and different clades) and several other sexually transmitted pathogens *in vitro* [27, 29, 33, 36, 45–50].

Contraceptive activity of PPCM is believed to be due to its ability to induce premature acrosomal loss; this effect requires physiological concentrations of extracellular Ca^2+^ [30]. Acrosomal integrity can be maintained in the presence of PPCM only in the absence of added extracellular Ca^2+^. It was important to demonstrate that PPCM retains its inhibitory activity against HIV in a Ca^2+^-independent manner. The present study has shown that the dose-response of PPCM-induced inhibition of HIV infectivity is independent of added Ca^2+^ (Figure 1).

Ideally, inhibition of HIV by PPCM would be irreversible, or at least pseudoirreversible. This allows for removal of all or most of the agent after viral inactivation, which, in turn, minimizes possible untoward side effects on cellular elements of the fertilization process and subsequent embryonic development. PPCM-induced viral inhibition is at least partially irreversible, since the dose-response of HIV inhibition is unaffected by a 1 : 100 dilution of PPCM after initial interaction with the virus (Figure 2). 

Not all mechanisms of HIV inhibition by PPCM are clearly understood. Part of the effect may be due to inhibition of viral entry. This occurs by preventing the interaction of the positively charged V3 loop of viral gp120 with negatively charged coreceptors (e.g., heparan sulfate or similar) on target (host) cells [51]. Studies with other polyanionic microbicides (e.g., cellulose sulfate, polystyrene sulfonate, cellulose sulfate phthalate) suggest that relatively high concentrations of these agents may cause gp41 six-helix bundle formation, viral disintegration and rapid loss of infectivity [52]. The loss of viral integrity suggests an irreversible mechanism of action. The work of Mesquita and coworkers [33] suggests possible irreversible inhibition of HIV-1 (BaL) and HIV-1 (RF) infectivity after pretreatment of immobilized virus with PPCM followed by washing, Further, a structural variant of PPCM inhibits HIV-1 (IIIB) and HIV-1 (BaL) in an irreversible or pseudoirreversible manner [51]. These data are in agreement with the present study.

 High concentrations of PPCM (25 mg/mL) are relatively noncytotoxic toward spermatozoa [27], as measured by a lack of effect on sperm motility following short-term exposure (Sander-Cramer test for spermicidal activity). We have confirmed and extended these studies by measuring sperm motility, forward progression and sperm viability after exposure to PPCM at concentrations as high as 10 mg/mL for periods up to 90 min (Figure 3). No effect was observed on sperm motility or forward progression. A small, but significant effect of duration of exposure on sperm viability was noted. The physiological significance of the latter finding is unclear, since no concentration-dependent effect was observed. The small effect of duration of exposure could reflect a small time-dependent decrease in viability, independent of the presence of PPCM. These data suggest that concentrations of and duration of exposure to PPCM that should be efficacious against HIV-1 have no substantial effect on sperm viability or selected aspects of sperm function.

 Earlier studies showing PPCM-induced acrosomal loss as a Ca^2+^-dependent process, dependent upon physiological concentrations of extracellular Ca^2+^ (ED_50_ approx. 0.08 mM), were carried out with PPCM at concentrations in the sub- *μ*g/mL to *μ*g/mL range [30]. The present study examined whether these observations could be extended to PPCM when used at concentrations several orders of magnitude higher (mg/mL range). It was important to evaluate whether previous exposure to PPCM affected subsequent ability of spermatozoa to undergo acrosomal loss in response to stimulus.

 Spermatozoa in Ca^2+^-deficient medium were pretreated with a high (10 mg/mL) concentration of PPCM, washed to reduce PPCM to nondetectable levels, and resuspended in Ca^2+^-replete medium. Under these conditions, acrosomal status is maintained at control levels in the absence of stimulus. Further, similar to that of control samples, PPCM-treated spermatozoa respond to acrosomal loss stimuli, namely calcium ionophore A23187 and PPCM (Figure 4). As a stimulus of acrosomal loss (in the presence of added extracellular Ca^2+^), PPCM was used at a concentration (0.25 *μ*g/mL) predicted to induce 50% maximal acrosomal loss [30]. Approximately 60% maximal loss was noted, in spermatozoa pretreated with PPCM as well as in control spermatozoa subjected to the same protocol (washing by centrifugation). The slight increase in acrosomal loss over that predicted may reflect an increased fragility of the acrosomes due to the experimental protocol of repeated centrifugation and resuspension. The data suggest that under appropriate conditions, prior treatment with relatively high concentrations of PPCM is without effect on acrosomal status.

 In order for PPCM-treated sperm to be used in ART procedures, it is important to establish that PPCM does not negatively affect sperm DNA integrity and/or sperm functions required for postfertilization restructuring of the sperm nucleus, once inside the egg. To test this, PPCM-treated sperm were analyzed with two tests: the Sperm DNA Fragmentation Assay (SDFA)/Sperm Chromatin Structure Assay (SCSA), a sperm structure assay used to measure DNA integrity [42]; and the Sperm DNA Decondensation (SDD) Test; a sperm function test used to measure overall sperm nuclear integrity [41]. Both the SCSA and SDD Test have clinical relevance in predicting live birth outcome [41, 42, 53]. Normal scores in PPCM-treated sperm for both tests support the use of PPCM-washed sperm in ART.

 The two tests were also used to test the hypothesis that the polyanionic nature of PPCM might allow the sperm nuclear membrane to be compromised through ionic interactions with either protamines or histones within the sperm chromatin. Protamine packaging greatly condenses the DNA, resulting in lower accessibility to DNA-damaging agents. If this is incomplete, exposed DNA may be vulnerable to damage from agents in the surrounding environment [54]. PPCM-induced interference with protamine-DNA interaction would be reflected as highly fragmented DNA, and/or delayed sperm DNA decondensation/nuclear swelling, as detected with the SDFA and SDD Test, respectively. However, PPCM (≤10 mg/mL) has no effect on either sperm DNA fragmentation (Figure 5(a)) or DNA decondensation (Figure 5(b)). These data are consistent with studies in which PPCM displayed no mutagenic activity, as measured by the Ames test [31]. Further, they support the safety of using PPCM-treated washed spermatozoa in ART to reduce risk of HIV infection.

 The ability of PPCM to inhibit HIV infectivity *in vitro* is well-documented [27, 28, 31, 49]. PPCM prevents HIV infection of several types of target cells *in vitro*. It also inhibits HIV transmission via dendritic cells [31]. The present study extended these studies by evaluating the ability of PPCM to inhibit HIV associated with whole semen.

Infected semen was simulated by incubating uninfected semen with a high titer of HIV-1 (BaL). This, rather than semen from HIV-infected men, was used to permit sufficiently high viral titers (particularly after washing by gradient centrifugation) to accurately quantify efficacy of PPCM combined with sperm washing beyond that effected by washing alone. With this method, viral titers were detectable even after 3 to 4-Log reductions.

Sperm washing by gradient and differential centrifugation reduced initial viral titer by somewhat over 90%, whether measured by viral p24 or RNA levels (Figures 6(a) and 6(b)). The method of sperm processing in the present study is similar to that used clinically by Peña and coworkers to reduce viral titers in semen from HIV-infected men [5]. The relatively inefficient removal of virus suggests that washing alone may not be an optimal method to completely remove high levels of viral contamination; this is also suggested by work presented in a review by Englert and coworkers [8]. These data are in agreement with other studies showing persistence of HIV in some samples after washing [4, 16]. 

Removal of soluble contamination by washing used in the present study is more efficient than removal of HIV. Concentration of PPCM, for example, is reduced from 10 mg/mL to less than 0.1 *μ*g/mL (limit of detection). Although the present study does not specifically address sperm binding of HIV, the data showing only a 90% reduction in viral titer by washing alone (Figure 6) are consistent with HIV binding to spermatozoa, described in other studies [17, 19, 20].

Treatment of HIV-spiked semen with PPCM substantially reduced viral titer. Levels of p24 in samples treated with PPCM, followed by washing by gradient and differential centrifugations were reduced by 3 Logs, as compared with p24 levels measured after washing alone (Figure 6(a)). 

Reduction of viral RNA (>4 Logs, as compared with washing alone) was greater than that of p24; RNA levels in the presence of PPCM were near the limit of detection of the assay. Since production of structural and regulatory RNA species precedes the production of structural proteins, these data suggest that RNA levels may be more sensitive than p24 levels to inhibition by at least some antiviral agents. In some instances, the release of p24 antigen can continue to occur even though HIV mRNA transcription and the production of infectious virus particles is inhibited [55]. Winters et al. [56] suggest that the use of proteins such as p24 to measure viral replication could obscure the effectiveness of an antiviral agent that primarily affects the transcription of certain types of viral RNA or DNA. Increased efficacy of PPCM when measured by viral RNA as compared with p24 measurements is consistent with mechanisms of inhibition of HIV infectivity in addition to inhibition of viral binding to target cells.

The present data show that PPCM can interact with and inactivate HIV associated with spermatozoa. Although these studies were restricted to a laboratory strain (BaL), results can likely be extended to other strains as well as clinical isolates, since PPCM is active against several HIV strains and isolates [28, 29, 31, 33]. Whether interaction of HIV with spermatozoa *in vitro* is similar to that observed in semen from HIV-infected individuals is unknown. Additional work is required to demonstrate efficacy of PPCM against sperm-associated HIV from HIV-infected men.

In spite of proven *in vitro* efficacy of polyanionic compounds against several sexually transmitted pathogenic microbes, including HIV-1, none has successfully completed Phase 3 clinical efficacy trials for HIV prevention [57–60]. Reasons for this apparent failure are not entirely clear. Part of the failure could be due to the trials themselves [58, 61]. 

Regardless of the reason for the failure of clinical trials, the efficacy of these compounds to prevent HIV and other microbial infections *in vitro* make them attractive candidates for reducing the risk of viral transmission from infected semen when processed *in vitro* for ART. The present study provides favorable data for the use of PPCM for this purpose. PPCM could also be useful for preventing transmission of other infections that coexist in HIV-infected individuals, including hepatitis C [62]. Other polyanions are active in preventing binding of hepatitis C to target cells [63], possibly by antagonizing interactions with heparan sulfate, or other heparin-like moieties on the cell surface [64].

Under appropriate conditions (i.e., use of Ca^2+^-deficient media, washing to remove compound after treatment prior to using Ca^2+^-replete media), PPCM can effectively reduce HIV infectivity by several orders of magnitude without adversely affecting sperm function. Although all data collected to date support the utility of PPCM as an antiviral prophylactic, additional work is clearly indicated to validate its use for ART. Successful validation and confirmation of lack of embryotoxicity of this method could create a new standard of care regarding the use of PPCM or other polyanionic microbicides to reduce infectivity of, or otherwise inactivate, one or more viruses (including HIV) that may be present in semen used in ART.

PPCM and other polyanionic compounds have broad-spectrum activities against a number of sexually transmitted pathogens (see Section 1). Our long-term goal will be to determine if PPCM can reduce the risk of transmission of one or more of several sexually transmitted microbes, in addition to HIV, when spermatozoa used for ART are routinely treated *in vitro* with this agent as part of a washing procedure based on data presented in the present study.

## 5. Conclusions

We examined the feasibility of improving the method of sperm washing used to reduce risk of HIV transmission during ART by adding PPCM, a microbicide for reducing sexually transmitted infections. A method is described in which PPCM, with sperm washing, is 3-4 orders of magnitude more efficacious than washing alone, in reducing HIV infectivity, without affecting sperm.
